# Electric-field-induced AFE-FE transitions and associated strain/preferred orientation in antiferroelectric PLZST

**DOI:** 10.1038/srep23659

**Published:** 2016-03-30

**Authors:** Teng Lu, Andrew J. Studer, Lasse Noren, Wanbiao Hu, Dehong Yu, Bethany McBride, Yujun Feng, Ray L. Withers, Hua Chen, Zhuo Xu, Yun Liu

**Affiliations:** 1 Research School of Chemistry, The Australian National University, ACT 2601, Australia; 2 Bragg Institute, The Australia Neutron Science and Technology Organisation, Lucas Height, Australia; 3 Electronic Materials Research Laboratory, Xian Jiaotong University, Xian 710049, Shaanxi, China; 4 Centre for Advanced Microscopy, The Australian National University, ACT 2601, Australia

## Abstract

Electric-field-induced, antiferroelectric-ferroelectric (AFE-FE) phase transitions are common for AFE materials. To date, the strain and preferred orientation evolution as well as the role of the intermediate FE state during the successive AFE-FE-AFE phase transitions has not been clear. To this end, we have herein studied a typical AFE Pb_0.97_La_0.02_(Zr_0.56_Sn_0.33_Ti_0.11_)O_3_ (PLZST) material using *in-situ* neutron diffraction. It is striking that the AFE-FE phase transition is not fully reversible: in the electric-field-induced FE state, the induced strain exhibits an elliptical distribution, which in turn leads to significant preferred orientation in the final AFE state after withdrawal of the applied electric-field. The *ω*-dependent neutron diffraction patterns show clear evidence of the induced strain distribution and associated preferred orientation arising from the AFE-FE phase transition. The current work also provides an explanation for several temperature and electric-field dependent dielectric anomalies as well as unrecovered strain change which appear in AFE materials after exposure to sufficiently high electric fields.

Antiferroelectricity (AFE) arises from anti-parallel alignment of off-center, ionic displacements in materials (of Pb^2+^ ions in the case of the first AFE compound to be discovered, PbZrO_3_^1^) and was initially proposed by Kittel in 1951^2^. The distinctive electromechanical coupling properties of AFE materials have attracted increasing attention ever since. A typical example is the lightly lanthanum-doped, lead zirconate stannate titanate (PLZST) system. At particular composition regions within this system, PLZST exhibits large electric-field-induced (E-field-induced) strain (*e.g*. maximum 0.87% for bulk ceramics and 0.49% for thin films^3^,^4^), potentially enabling a range of device applications, such as actuators, sensors, electrocaloric devices, energy harvesting and storage devices^4^,^5^,^6^,^7^. Such high strains have long been considered to be related to a reversible, E-field-induced, tetragonal antiferroelectric (AFE_T_) to rhombohedral ferroelectric (FE_R_) phase transition^8^. That the initial physical properties of fresh samples behave differently to those of poled samples suggests that the AFE_T_-FE_R_ transition may not be fully reversible. Likewise, the fact that the physical properties of PLZST as a function of increasing E-field are initially linear but then exhibit clear hysteresis behaviour^9^ as well as a strong field and/or temperature dependence, again suggests that a simple reversible AFE_T_-FE_R_ transition is not the whole story.

Park *et al*.^10^ claimed a decoupling of the transverse and longitudinal strain changes accompanying the AFE_T_ to FE_R_ phase transition, and attributed it to preferentially oriented AFE domains induced by the externally applied electric field. The X-ray diffraction (XRD) study used, however, had limitations in (1) the lack of any direct *in-situ* characterisation evidence for changes in the AFE domain configuration with applied field, including the formation of preferentially oriented AFE domains and their associated dynamic behavior; and (2) the XRD patterns were only collected before and after exposure to an E-field of 50 kV/cm, much higher than the critical field (37 kV/cm) for AFE-FE phase switching. It is thus unclear what exactly happens for samples at lower fields before and after the phase transition. Furthermore, how do the average structure and the preferred orientation relate to one another? And to what extent does such preferred orientation affect the measured physical properties? These questions need to be answered in order to guide the development and optimization of AFE materials of this type for practical applications.

In this work, *in-situ* neutron powder diffraction is employed to investigate the evolution of preferred orientation and strain in a Pb_0.97_La_0.02_(Zr_0.56_Sn_0.33_Ti_0.11_)O_3_ (PLZST in what follows) sample as a function of applied E-field. Note that the fresh PLZST sample with the chemical composition just given is in the AFE_T_ tetragonal phase region, as reported previously^3^,^8^. In conjunction with dielectric, ferroelectric properties measurements, we aim to attain a comprehensive understanding of the E-field-induced, AFE_T_ (initial)- > FE_R_- > AFE_T_ (final) phase transition and the associated impacts on properties, thereby providing new insight to the design and development of AFE materials.

## Results and Discussion

### *In-situ* neutron powder diffraction

**Figure 1:**
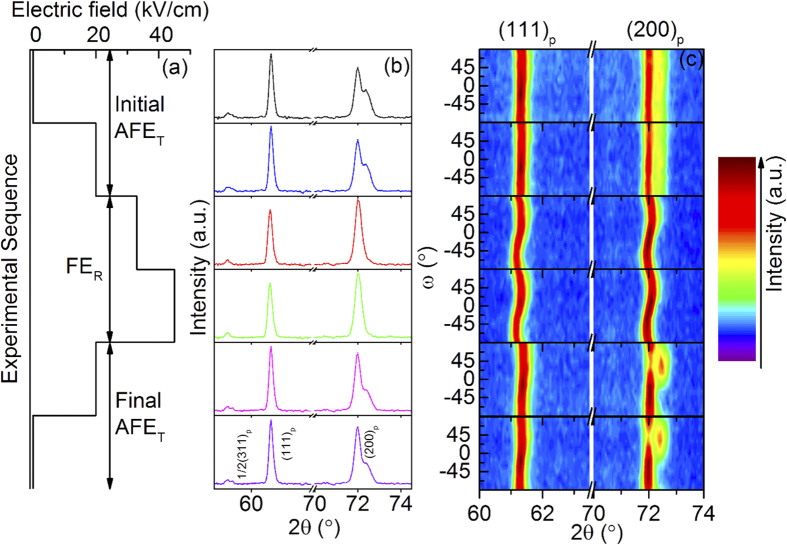
*In-situ* neutron diffraction patterns (NDPs) collected at room temperature. (**a**) A schematic drawing to describe the experimental sequence of applied E-fields, resulting in three different states *i.e*. the initial AFE_T_ state, the intermediate FE_R_ state and the final AFE_T_ state, determined by their average NDPs partially presented in (**b**) (57.5° < 2*θ* < 74.5°). (**c**) The *ω*-dependence of the (111)_p_ and (200)_p_ peaks. Both the *ω*-averaged NDPs (**b**) as well as the *ω*-dependent NDPs (**c**) were collected *in-situ* under the applied E-fields shown in (**a**).

Detail as regards sample preparation, *in-situ* neutron powder diffraction under applied E-field (including experimental setup and procedures followed) is presented in the “Methods” section below. Fig. 1a shows the experimental sequence of external E-fields applied (0 kV/cm → 20 kV/cm → 33 kV/cm → 45 kV/cm → 20 kV/cm → 0 kV/cm). Fig. 1b shows the corresponding 57.5° < 2*θ* < 74.5° regions of the *ω*-averaged, neutron diffraction patterns (NDPs) obtained as a function of E-field. Note that the NDPs in (b) were summed and averaged over the 13 *ω*-dependent patterns, collected at rotation angles from *ω* = −90° to + 90° in incremental steps of 15°. Fig. 1c maps the *ω-*dependence of the characteristic (111)_p_ and (200)_p_ peaks, subscript p for the underlying parent perovskite structure.

All patterns (Fig. 1b,c) are indexed with respect to the underlying parent perovskite structure (labelled with the subscript p). Note that when the applied E-field is below 33 kV/cm, the shape of the (111)_p_ peak appears symmetric while the (200)_p_ peak is clearly split into two peaks with a relative intensity ratio of ~2:1 (Fig. 1b), consistent with pseudo-tetragonal metric symmetry, as expected. In addition, distinct ½(111)_p_ (not shown in Fig. 1b) and split ½(311)_p_ type satellite reflections are also observed, in good agreement with our own electron diffraction results (Fig. S1a) as well as those previously obtained from similar composition samples^11^,^12^. Satellite reflections of this type are highly likely to be associated with *a*^*−*^*a*^*−*^*c*^0^ ZrO_6_ octahedral tilting as also occurs in PbZrO_3_ itself, but not directly relevant to the Pb ion displacements responsible for the AFE structure^11^. Indeed, Íniguez *et al*.^13^ have recently shown that *a*^*−*^*a*^*−*^*c*^0^ ZrO_6_ octahedral tilting is essential to the stabilization of the AFE structure of PbZrO_3_. Given that our PLZST sample can be thought of as being composed of PbZrO_3_ (56%), PbSnO_3_ (33%) and PbTiO_3_ (11%), it should be expected that the same *a*^*−*^*a*^*−*^*c*^0^ pattern of octahedral rotation characteristic of the dominant PbZrO_3_ component also occurs in our sample). In the case of PbZrO_3_, the pattern of off-centre Pb ion displacements giving rise to its AFE structure is associated with the modulation wave-vector **q** = ^1^/_4_ [110]_p_^*13^. In the case of PLZST and related samples, it is well known that the equivalent single-**q**, primary modulation wave-vector is of ^1^/_*n*_ [110]_p_* type^11^,^12^,^14^ where the value of *n* is larger than 4 and dependent on composition. In our case, *n* was ~10 via electron diffraction (Fig. S1b). Such satellite reflections, however, were not observed in the NDPs.

Considering our focus in this work is the E-field-induced evolution of the average structure and associated phenomena, the NDPs of the AFE_T_ phase at this stage were therefore described as a pseudotetragonal phase with *a* = *b* = 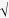
*2a*_*p*_ and *c* = *2c*_*p*_, where the corresponding primitive, parent perovskite, unit-cell parameters were refined to be *a*_*p*_ = *b*_*p*_ > *c*_*p*_ [*a*_*p*_ = 4.1087(5) Å and *c*_*p*_ = 4.0870(5) Å] to accommodate octahedral tilting. Note that the following discussion is mainly based on the parent perovskite structure. With an increase in the applied E-field up to, or beyond, 33 kV/cm, note that the (200, 020)_p_/(002)_p_ and ½(311)_p_/½(113)_p_ doublet peaks appear to merge together while the (111)_p_ peak remains unsplit, suggesting the formation of a new phase of pseudocubic metric symmetry. It is clear that the new phase is ferroelectric (FE) from the E-field dependent *P*-E loops. The previous *in-situ* XRD study^8^ of a similar PLZST sample with higher resolution showed that the high field phase is indeed a FE rhombohedral structure, although very close to metrically cubic. The refined rhombohedral lattice parameters (using the whole NDP pattern) under this assumption are *a*_*R*_ = 4.1070(5) Å and *α* = 89.93(3)°, in the rhombohedral setting. The continued presence of observable ^1^/_2_(111)_p_ and ½(311)_p_ satellite reflections is not inconsistent with *a*^*−*^*a*^*−*^*a*^*−*^ octahedral tilting^15^,^16^, indicating a rhombohedral *R3c* space group symmetry. This AFE_T_-FE_R_ phase transition occurs when the applied E-field becomes sufficiently large. With decreasing E-field from the maximum applied field of 45 kV/cm, the PLZST sample reverts, as expected, to the AFE_T_ phase.

In terms of the NDPs shown in Fig. 1b,c, the sample can be classified into three states: the initial AFE_T_, the intermediate FE_R_ and the final AFE_T_ states. The *ω*-averaged, NDPs of the initial and final AFE_T_ states (Fig. 1b) suggest that the field-induced AFE-FE phase transition is quite reversible. The *ω*-dependent NDPs in Fig. 1c, however, clearly demonstrate an irreversible change in the preferred orientation, or texture, of the AFE_T_ phase on cycling through the phase transition. The fact that the *ω*-dependent NDPs collected at 0 kV/cm and 20 kV/cm, respectively, are almost identical showing that insufficiently strong, applied E-fields have minimal impact on preferred orientation in the AFE_T_ state.

Once the applied E-field becomes sufficiently strong, however, it induces the phase transition from the AFE_T_ to the FE_R_ state signified by the (200)_p_ reflections merging into a single peak while the (111)_p_ peak remains unaffected. Note that the exact diffraction peak positions in the FE_R_ state now depend strongly on *ω*, showing a characteristic *S*-shape trajectory (Fig. 1c). With further increase in the E-field within the FE_R_ region of stability, note that the *ω*-dependent NDPs again show no obvious change. Upon reducing the E-field sufficiently to undergo the transition back to the final AFE_T_ state, note that the *ω*-dependence of the (200)_p_ reflection doublet now exhibits very strong texture, by contrast with the initial AFE_T_ state. As can be seen in the bottom two panels of Fig. 1c, starting from the + *ω* side (in the vicinity of 90°), the intensity of the doublet (200, 020)_p_ peak (on the low angle side) is strong while that of the (nominally 50% weaker) (002)_p_ peak is barely visible. Upon decreasing + *ω* to 30°, however, the relative intensities of the split (200, 020)_p_ and (002)_p_ peaks are now much closer to 1:1. The (002)_p_ peak on the high angle side becomes much stronger and broader, also separating itself quite significantly from the (200, 020)_p_ peak on the low angle side. Upon further decreasing *ω* to the −*ω* side, the intensity of the (002) peak gradually disappears until *ω* = *−*60° when it becomes barely observable again. From these *in-situ* NDPs, it is also clear that the *ω*-dependent NDPs are independent of applied E-field within the region of stability of the AFE_T_ state *i.e*. before and after the phase transition, the 20 kV/cm change in external field has little impact on the *ω*-dependent NDPs. This result differs with Park *et al*,’^10^ suggestion that the formation of preferred orientation within the AFE_T_ state occurs as soon as an E-field is applied. Our results show that it is the E-field-induced phase transition that has a quite considerable impact on the texture of the PLZST samples.

Preferred orientation implies a non-random distribution of grain orientations and/or domains within the pellet. For the AFE_T_ state, it is well established that changes in the intensity ratio of the low angle (200, 020)_p_ peak to the high angle (002)_p_ peak reflect any such preferred orientation and are clearly present only in the final AFE_T_ state (see the bottom 2 panels of Fig. 1c). This is often ignored for reversible AFE-FE phase transitions, although not in the work of Park *et al*.^10^. Within the FE_R_ state, the observed variation in peak position as a function of *ω* implies changes in internal and/or intergranular strain for ferroelectric materials under an applied E-field^17^,^18^,^19^. In order to quantify the observed evolution in preferred orientation, the parameter *f*_200_(MRD)^17^ (MRD = Multiple of Random Distribution), proportional to the relative domain fractions along the *a*_p_ and *c*_p_ directions, is used as an index and calculated as follows:


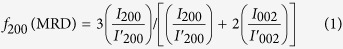


where *I* denotes the peak intensity, calculated by multi-peak fitting of the final AFE_T_ NDPs using two pseudo-Voigt profiles, while *I’* is defined as the peak intensity for the sample with random orientation. Note that the *I’* value here was approximated by summing and averaging the observed intensities over all 13 measured *ω* values. (It is not available to measure the polycrystalline sample (a pellet here) with random orientation after the application of a high voltage)^20^.

**Figure 2:**
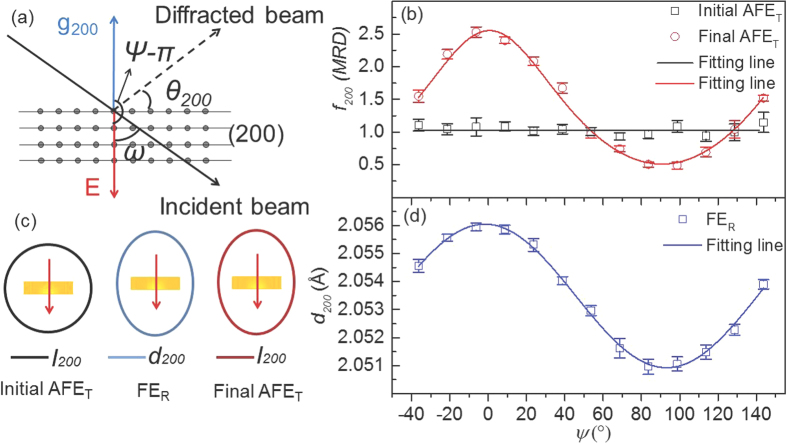
*ψ*-dependence of the strain and associated preferential orientation evolution during the E-field induced AFE-FE phase transition. (**a**) A schematic drawing of the neutron diffraction geometry, especially for the (200) lattice plane, describing the relationship between *ψ* and *ω. ψ* is the angle between the diffraction vector, **g**_hkl_, perpendicular to the (*hkl*) planes and the applied field **E,** modulo 180° in this neutron diffraction setup. (**b**) *f*_*200*_*(MRD)* that proportionally reflects the domain fraction along *a*_p_ and *c*_p_ directions, as a function of *ψ* for the initial and final AFE_T_: *f*_*200*_*(MRD)* of the initial AFE_T_ state remains unchanged and for the final AFE_T_ it can be fitted by the function (combining elliptical function of *I*_200_ with *f*_*200*_*(MRD)* calculation formula) with the maximum value appeared at the *ψ* value of ~0°. (**c**) The schematic diagrams demonstrate how the *d*_*200*_ and *I*_*200*_ vary during the AFE-FE phase transition (distance from the centre of the pellets denotes the amplitude): the *I*_*200*_ is isotropic, regardless of the E-field in the initial AFE_T_ state (black line); in the FE_R_ state, the *d*_*200*_ expands along the applied E-field and contracts along the direction perpendicular to the field direction and in the final AFE_T_ state, the *I*_*200*_ exhibits the similar preferred orientation with *d*_*200*_ in the FE_R_ state. (**d**) The *d*_*200*_ value changes as a function of *ψ* and the associated curve can be fitted by an elliptical function with the maximum value approximately at *ψ* = 0° and 180° and the minimum value at *ψ* = 90°.

In order to clearly demonstrate the appearance of preferred orientation, the azimuthal angle, *ψ*, was used instead of the rotation angle *ω* for further analysis. The parameter *ψ* is the angle between the diffraction vector, **g**_hkl_, of the (*hkl*) plane and the applied field **E**, modulo 180°. In general, the relationship between *ψ* and *ω* is: *ψ* = *ω–θ*_hkl_ + 90°, where *θ*_hkl_ is the Bragg angle for the particular (*hkl*) reflection. For the (200)_p_ peak in particular, *θ*_200_ ≈ 36°, as shown in Fig. 2a. The *f*_200_ (MRD) curves for the initial and final AFE_T_ states with respect to *ψ* are shown in Fig. 2b. For the initial AFE_T_ state, the intensities, and hence *f*_200_(MRD) for the (200)_p_ reflections, remains constant at all values of *ψ* (see the black line in Fig. 2b), indicating that samples in the initial AFE_T_ state are isotropic *i.e*. there is no observable preferred orientation, or texture (see the black lines in Fig. 2c).

By contrast, in the final AFE_T_ state, the calculated *f*_200_(MRD) versus *ψ* curve has a distinct *S*-shape profile, indicating clear preferred orientation. Note, however, that there is no obvious strain *i.e*. no obvious change in the *d*_200,020_ or *d*_002_ peak positions (*cf*. the bottom 2 panels of Fig. 1c). To further understand the mechanism underlying such E-field-induced changes, it is essential to link the behaviour of the initial AFE_T_ state to that of the intermediate FE_R_ state to the final AFE_T_ state.

In the intermediate FE_R_ phase, the originally split (200)_p_ peaks merge together. The *d*_200_ spacing for this single peak, however, now varies strongly with *ψ*, as shown in Fig. 2d. It is well accepted in the literature that this *d*_200_ spacing change is primarily responsible for observed macroscopic elastic strain during the poling process in rhombohedral ferroelectric (FE) materials, assuming that E-field-induced domain switching does not need to be taken into account^18^. The measured *d*-*ψ* curve can in fact be well fitted by the following elliptical function


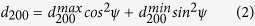


where 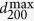 = 2.0559(1) Å at *ψ* = 0 ± 1° and 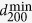 = 2.0509(2) Å at *ψ* = 90 ± 1°. Note that the maximum *d*_200_ value occurs when the **g**_200_ diffraction vector, perpendicular to the (200) planes, is parallel to the applied E-field, indicating the sample in the FE_R_ state exhibits a strong expansion along the direction parallel to the E-field. Similarly, the minimum value at *ψ* = 90° implies an associated contraction along the direction perpendicular to the E-field *i.e*. parallel to the surface of the pellets. Using this fitting function, the distribution of *d*_200_ within the pellet *i.e*. the elastic strain can be schematically depicted in terms of an elliptical shape (see the blue line in Fig. 2c).

Once the sample enters into the final AFE_T_ state, the single (200)_p_ peak in the FE_R_ state splits back into a (200, 020)_p_ and (002)_p_ doublet. Because the sum of the measured *I*_200,020_ and *I*_002_ intensities remains constant after normalising the overall intensity of the NDPs, *i.e., I*_200,020_ + *I*_002_ = constant, so *f*_200_(MRD) can be simply calculated by using *I*_200_ alone. It is well known that preferred orientation is a consequence of the minimization of overall energy. The elastic strain observed in the FE_R_ state should thus be very closely related to the preferred orientation observed in the final AFE_T_ state. Experimentally, *I*_200_ follows the same form as the elliptical function given above for *d*_200_ (eq. 2). Thus the *f*_200_(MRD) versus *ψ* distribution of the final AFE_T_ state is indeed well fitted by a function that replacing elliptical function *I*_200_ into the eq. 1 (see the red line in Fig. 2b). The maximum and minimum of *f*_200_(MRD) appear at exactly the same *ψ* values as that found for *d*_*200*_ in the FE_R_ state. Obviously, the close relationship between *d*_*200*_ in the FE_R_ state and *f*_200_(MRD) in the final AFE_T_ state further supports the contention that the elastic strain observed in the FE_R_ state is the main driving force for the preferred orientation distribution when the sample returns from the FE_R_ state to the final AFE_T_ state.

Clearly the texture of the final AFE_T_ state is not the same as that of the initial AFE_T_ state and is strongly dependent on the applied E-field history of the particular sample. In the initial AFE state, PLZST exhibits a pseudo-tetragonal average structure and no preferred orientation. Likewise, there is still no preferred orientation when the applied E-field, *e.g*. at 20 kV/cm, is insufficient to induce the AFE_T_ to FE_R_ transition. This result is different to the prediction made by Park *et al*.^10^, where it was suggested that the initial, randomly oriented AFE domains would become preferentially oriented AFE domains under an applied field. Once the E-field is high enough to induce the AFE_T_-FE_R_ phase transition, considerable strain change appears in the intermediate FE_R_ state. The strain is no longer homogeneous as a consequence of the elongation of the rhombohedral unit cell in the direction along the applied E-field and its contraction in the direction perpendicular to this field (see the black line in Fig. 2c). This results in an elliptical distribution of *d*_200_ (proportional to the structural strain) as a function of *ψ* (the middle panel of Fig. 2c). This phase transition induced strain is again E-field independent as long as the FE_R_ phase remains. Upon the reverse phase transformation, clear preferred orientation in the final AFE_T_ state becomes apparent and appears to be directly determined by the strain distribution in the intermediate FE_R_ state (*cf. e.g*. Fig. 2b,d), *i.e*. the *a*_*p*_-axis of the pseudotetragonal cell prefers to align parallel to the E-field while the *c*_*p*_-axis aligns perpendicular to the E-field (see the red line in Fig. 2c). This preferred orientation in the final AFE_T_ state is again independent of the applied weak E-field as long as the final AFE_T_ state remains. Such strain driven preferred orientation was also reported in pre-poled FE materials^21^, in reasonable agreement with our conclusions.

### Electric properties

It now becomes interesting to investigate how this texture evolution affects measured physical properties. We have thus also investigated the dielectric, ferroelectric and antiferroelectric properties change of the PLZST samples after exposure to different E-field strengths. All measurements have been carried out using fresh samples (labelled PLZST0) as well as samples which have been exposed to applied E-fields of 20, 33 and 45 kV/cm only once (labelled PLZST 20, 33 and 45 respectively), in order to avoid any influence from repeated high electric field exposure on the texture of the material.

**Figure 3:**
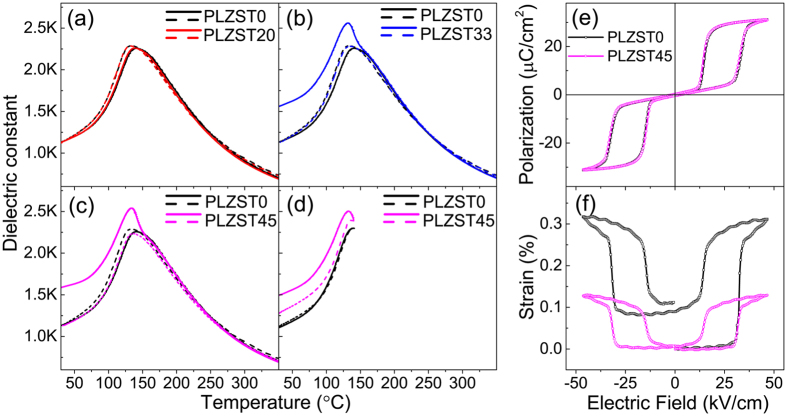
Dielectric and ferroelectric properties of the PLZST ceramics after applying different E-fields. Temperature-dependent dielectric spectra of the samples after applying (**a**) 20, (**b**) 33 and (**c**) 45 kV/cm field, measured during the heating (solid line) and cooling (dashed line) in the temperature range of 30–350 °C, where the black solid and dashed lines curves are measured from the fresh sample for the reference. (**d**) Temperature-dependent dielectric spectra of the PLZST0 and PLZST45 measured in the temperature range from the room temperature to 140 °C. The first cycle of *P–E* (**e**) and *S-E* (**f**) hysteresis loops of PLZST0 and PLZST45 are measured at 1 Hz.

Fig. 3 shows the measured temperature-dependent dielectric spectra collected from samples after exposure to the designated E-fields. The dielectric constant of the PLZST0 sample exhibits a plateau region over the temperature range 131–160 °C. This has previously been attributed to the multi-cell cubic (MCC) state existing in PZST-based AFE materials^22^,^23^, a consequence of the Sn substitution that disrupts the antiferroelectric-paraelectric transformation. In addition, thermal dielectric hysteresis was observed and the dielectric maximum occurs at lower temperature for the cooling process, indicative of the nature of a first-order phase transition^24^. Although dielectric plateaus are observed in all PLZST samples, their temperature-dependent dielectric properties differ. PLZST20 (Fig. 3b) shows very nearly the same dielectric behaviour as PLZST0 on both heating and cooling. This is attributed to the fact that the average structures and *a/c* domain configurations of PLZST20 are very similar to those of PLZST0, as also indicated by the *in-situ* NDPs and the fact that the AFE-FE phase transition has not yet been triggered when the applied E-field is below 33 kV/cm. In comparison, the PLZST33 (Fig. 3c) and PLZST45 (Fig. 3d) samples exhibit a relatively sharp dielectric peak around 131 °C and a significantly higher dielectric constant than PLZST0 and PLZST20 during the heating process. By contrast, on cooling, both samples revert to the same trend behaviour as the PLZST0 sample *i.e*. a broadened dielectric peak and a reduced magnitude in the maximum dielectric constant.

The above dielectric phenomena are clearly correlated with the presence or absence of preferred orientation and associated strain in the starting samples for dielectric measurement *i.e*. with their applied E-field history prior to the temperature-dependent, dielectric measurements. The different dielectric behaviour of the PLZST33 and PLZST45 samples for dielectric measurements upon *e.g*. heating can be explained by the preferred orientation and associated strain induced by the prior, E-field-induced, AFE-FE phase transition, as discussed above. As the E-field is sufficient to induce the intermediate FE stage, the strain in the FE state drives the formation of preferred orientation *e.g*., *a*_*p*_-axis prefers align parallel to the E-field at the final AFE stage. Such a realignment is likely to enhance the dielectric constant measured in the same direction. The preferred orientation in the final AFE_T_ state thus significantly enhances the dielectric constant for both the PLZST33 and PLZST45 samples.

On the other hand, the fact that the dielectric constant behaviours of the PLZST33 and PLZST45 samples exhibit no obvious change by comparison with the PLZST0 sample during the cooling process is equally interesting. Note that the cooling temperature process starts from the paraelectric (PE) state at 350 °C, far above T_max_, the temperature point where the dielectric constant is at a maximum. The initial preferred orientation would thus have disappeared in order to minimize the free energy. The dielectric constant therefore remains the same as the fresh sample during the cooling process. If, however, the temperature is only increased to 140 °C for 40 mins on heating and then dropped back down to room temperature, it is found that the dielectric constant of PLZST45 (Fig. 3d), during the cooling cycle, is still slightly larger than that of PLZST0 measured under the same conditions since the some degree of preferred orientation still remains at 140 °C. It is suggested that when heating PLZST up to the temperature range of the dielectric plateau, some of the AFE phase transforms into the PE phase. This phenomenon indirectly supports the statement that the MCC region formed as a result of Sn doping disrupts the antiferroelectric-paraelectric transformation.

The polarisation-field and strain-field hysteresis loops (*P-E* and *S-E*, respectively) of the PLZST0 and PLZST45 samples are presented in Fig. 3e,f. It is found that both samples have the same double *P-E* hysteresis loops but the strain-field hysteresis loops are quite different for the two samples. The PLSZT0 sample shows a large strain variation (0.32%) and does not return to its original point (the zero point) after the first cycle. This can again be explained by the AFE_T_-FE_R_ phase transition induced irreversible strain and associated preferential orientation as abovementioned. The PLZST45 sample, on the other hand, shows a small strain change (~0.14%) when the same E-field is applied and its strain loop returns to its original point at 0 kV/cm. This can be attributed to the reversible part of the strain evolution during the AFE-FE phase transition occurring in this material. This result explains why the first and second strain hysteresis loops are never the same in this type of AFE materials, such as PLZST in this work and Pb_0.99_Nb_0.02_[(Zr_0.57_Sn_0.43_)_0.94_Ti_0.06_]_0.98_O_3_^25^.

## Conclusions

In summary, the results from an *in-situ* neutron powder diffraction investigation of Pb_0.97_La_0.02_(Zr_0.56_Sn_0.33_Ti_0.11_)O_3_ show that an applied E-field not only induces an AFE_T_-FE_R_ phase transition but also produces a significant elliptical strain distribution in the FE_R_ state as well as corresponding preferred orientation in the final AFE_T_ state obtained upon withdrawal of the E-field. The strict reversibility of the phase transition and recovery of the initial AFE_T_ phase after the withdrawal of the E-field is thus called into question. As a result, the dielectric polarisation of AFE materials is enhanced after exposure to sufficiently high E-fields. It also explains the different strain behaviour between the first and second strain-field cycles of such materials.

We believe this work provides a complete picture to describe the micro-structure and properties of antiferroelectric PLZST ceramics. It also provides critical experimental evidence correcting Park *et al*.’s^10^ original prediction on E-field-induced preferred orientation in the initial AFE phase. The approach used in this work to directly observe the strain and preferred orientation during the E-field-induced AFE_T_-FE_R_ phase transition and associated mechanism may also be applicable to other kinds of materials with similar behaviour, indicating a new method for designing multi-functional composites via interface strain transfer. Furthermore, the current work suggests that to achieve a high and reversible strain in AFE materials, the operating E-field needs to be optimized. It also paves the way to minimize irreversible factors to improve the energy recovery and storage capabilities of AFE materials when used for energy storage applications.

## Method

### Sample preparation

Powder samples of Pb_0.97_La_0.02_(Zr_0.56_Sn_0.33_Ti_0.11_)O_3_ were synthesized by conventional solid state reaction, following the procedure outlined in the reference^26^, before being pressed into ceramic pellets. All sample pellets were then heat-treated at 300 °C for 6 hrs in order to release residual stress resulting from the manufacturing process before characterizing the structure and properties. Electrodes were applied by coating silver paint onto the pellet surfaces and then heat-treating them at 550 °C to achieve good electrical contact.

### *In-situ* neutron diffraction

**Figure 4:**
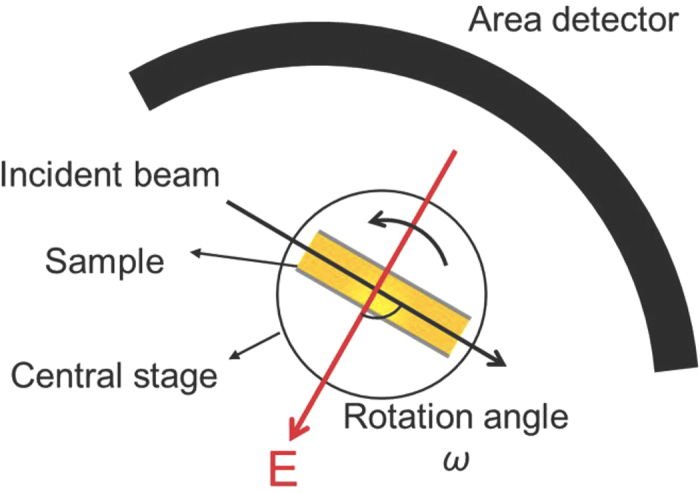
The schematic of the setup of the *in-situ* neutron diffraction experiment. Yellow pellet denotes PLZST ceramic sample with Ag electrode (shown as grey lines). The rotation angle *ω* was initially set to −90°.

*In-situ* neutron diffraction patterns were collected at a wavelength of 2.41 Å using WOMBAT, the high-intensity powder diffractometer installed on the Opal reactor of the Bragg Institute at the Australian Nuclear Science and Technology Organisation^27^. The *in-situ* neutron diffraction set-up used is shown in Fig. 4 and the collection strategy is same as the one used by Wang *et al*.^28^ and Simons *et al*.^29^. A sample holder was used to fix the sample pellet to a central stage which could then be rotated around the vertical *ω* axis. Conducting wires were attached to the silver coatings on either side of the pellet to enable a voltage to be applied. The external E-field was always applied normal to the ceramic pellet surface using the E-field sequence 0 kV/cm → 20 kV/cm → 33 kV/cm → 45 kV/cm → 20 kV/cm → 0 kV/cm. At each field point, the sample was initially set so that the applied field was orthogonal to the incident beam (*i.e*. at *ω* = −90°, where *ω* is the rotation angle between the incident beam and the applied E-field). The sample stage was then rotated anticlockwise from *ω* = −90° to + 90° in-plane at increments of 15°. In total, 13 patterns were therefore collected for each individual E-field.

### Electrical properties

The ceramic pellet samples with silver electrodes were exposed to an E-field of 20, 33 or 45 kV/cm for 15 min at room temperature in a silicone oil bath. The temperature dependent-dielectric constants were measured at 10 kHz using a high precision inductance, capacitance and resistance meter (LCR meter, Agilent 4980 A). The polarization-field (P-E) and strain-field (S-E) hysteresis loops were investigated using an aixACCT FE test unit in conjunction with a laser interferometer.

## Additional Information

**How to cite this article**: Lu, T. *et al*. Electric-field-induced AFE-FE transitions and associated strain/preferred orientation in antiferroelectric PLZST. *Sci. Rep*. **6**, 23659; doi: 10.1038/srep23659 (2016).

## Supplementary Material

Supplementary Information
