# Supplementary material for: Electric-field-induced AFE-FE transitions and associated strain/preferred orientation in antiferroelectric PLZST
Source: Sci Rep. 2016 Mar 30;6:23659. doi: 10.1038/srep23659 (PMC4812248; doi:10.1038/srep23659)
Supplement: Supplementary Information [file srep23659-s1.pdf]

Electric-field-induced AFE-FE transitions and associated strain/preferred orientation in antiferroelectric PLZST

Teng Lu[1], Andrew J. Studer[2], Lasse Noren[1], Wanbiao Hu[1], Dehong Yu[2], Bethany McBride[1], Yujun Feng[3], Ray L.Withers[1], Hua Chen[4], Zhuo Xu[3] and Yun Liu[1,*]

[1] Research School of Chemistry, The Australian National University, ACT 2601, Australia

[2] Bragg Institute, The Australia Neutron Science and Technology Organisation, Lucas Height, Australia

[3] Electronic Materials Research Laboratory, Xian Jiaotong University, Xian 710049, Shaanxi, China

[4] Centre for Advanced Microscopy, The Australian National University, ACT 2601, Australia

*Correspondence to: yun.liu@anu.edu.au.

The electron diffraction patterns of the PLZST samples

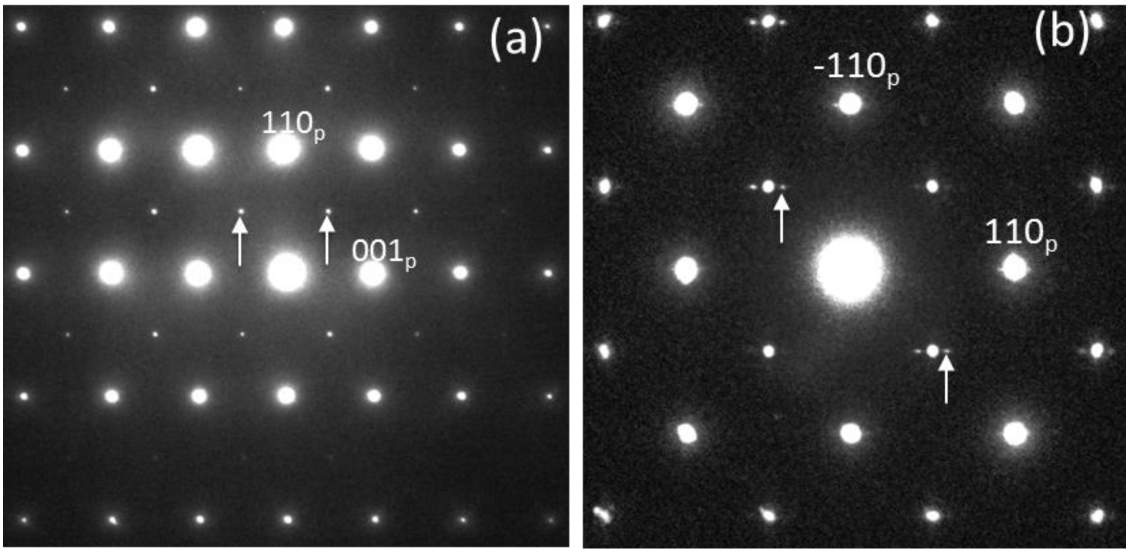

Figure S1 (a) the <1-10>$_P$ and (b) <001>$_P$ zone axis electron diffraction patterns (EDPs) of the PLZST sample. It shows clearly the G$\pm$1/2[111]*$_P$ satellite diffraction spots which is possibly related to the octahedral tilting. In addition, although the pattern is

not so clear, the $1/n[110]^*_p$ type incommensurate spots are unambiguous and n was ~ 10 in our case.
